# Processing Optimization and Characterization of Angiotensin-Ι-Converting Enzyme Inhibitory Peptides from Lizardfish (*Synodus macrops*) Scale Gelatin

**DOI:** 10.3390/md16070228

**Published:** 2018-07-04

**Authors:** Junde Chen, Ying Liu, Guangyu Wang, Shanshan Sun, Rui Liu, Bihong Hong, Ran Gao, Kaikai Bai

**Affiliations:** 1 Marine Biological Resource Comprehensive Utilization Engineering Research Center of the State Oceanic Administration, the Third Institute of Oceanography of the State Oceanic Administration, Xiamen 361005, China; ly6049787@163.com (Y.L.); 17859733637@163.com (G.W.); shshsun123@163.com (S.S.); bhhong@tio.org.cn (B.H.); rgao@tio.org.cn (R.G.); kkbai@tio.org.cn (K.B.); 2 Jiangsu Collaborative Innovation Center of Chinese Medicinal Resources Industrialization, and National and Local Collaborative Engineering Center of Chinese Medicinal Resources Industrialization and Formulae Innovative Medicine, Nanjing University of Chinese Medicine, Nanjing 210023, China; cpulr@126.com

**Keywords:** gelatin hydrolysates, ACE inhibitory peptides, response surface methodology, lizardfish scales, nano LCMS/MS

## Abstract

Hypertension can cause coronary heart disease. Synthetic angiotensin-converting enzyme (ACE) inhibitors are effective antihypertensive drugs but often cause side effects. The aim of this study was to prepare potential ACE inhibitors from scales. Gelatin was extracted from lizardfish scales. Then, scale gelatin was enzymolyzed to prepare ACE inhibitory peptides using response surface methodology. Proteolytic conditions after optimization were as follows: pH 7.0, enzyme substrate ratio 3.2%, temperature 47 °C, and proteolysis lasting 2 h and 50 min. The experimental ACE inhibitory activity under optimal conditions was 86.0 ± 0.4%. Among the 118 peptides identified from gelatin hydrolysates, 87.3% were hydrophilic and 93.22% had a molecular weight <2000 Da. Gelatin peptides had high stability upon exposure to high temperature and pH as well as gastrointestinal tract enzymes. Gelatin peptides showed an antihypertensive effect in spontaneously hypertensive rats at a dosage of 2 g/kg in the long-term experiments. A new ACE inhibitory peptide was isolated from gelatin hydrolysates, and was identified as AGPPGSDGQPGAK with an IC_50_ value of 420 ± 20 μM. In this way, ACE inhibitory peptides derived from scale gelatin have the potential to be used as healthy ACE-inhibiting drug raw materials.

## 1. Introduction

Hypertension can cause cardiovascular, cerebrovascular, and renal diseases, as well as stroke [1]. Angiotensin-converting enzyme (ACE, 3.4.15.1) plays a crucial role in regulating blood pressure by converting angiotensin І to the octapeptide angiotensin II, a potent vasoconstrictor that is responsible for the development of hypertension, and by degrading the vasodilator bradykinin [2]. Therefore, the inhibition of ACE has been considered the first-line target in the prevention and the treatment of hypertension [3]. During the past two decades, a series of ACE inhibitors, such as benazepril, captopril, and perindopril, have been employed as effective synthetic antihypertensive drugs, but they often cause side effects such as coughing, skin rashes, and taste disturbances [4,5,6]. For these reasons, food-derived ACE inhibitory peptides have attracted increasing attention, since they are safer and healthier than synthetic drugs. Several ACE inhibitory peptides have been isolated from various food resources, such as sardinelle muscle [7], thornback ray skin [2], and jellyfish [8]. Katsuobushi oligopeptide, a protein hydrolysate that is digested by thermolysin from dried bonito, has been approved as a “Food for Specified Health Use” by the Ministry of Health and Welfare of Japan, where it is marketed for the treatment of hypertension [9].

Wild-caught fish are important foods, accounting for approximately 50% of the total world fish production. More than 70% of this product is subjected to processing [10,11,12]. During processing, a large amount of scales is produced, accounting for approximately 2% of total fish weight [13]. These scales are rich in proteins that can release ACE inhibitory peptides by proteolysis [14,15]. The lizardfish (*Synodus macrops*) is widely distributed in tropical and subtropical waters. It is one of the most commercially important fish in China. Unfortunately, processing plants dump lizardfish scales as waste. The preparation of active peptides from lizardfish scales would not only improve the added value of fish products, but also reduce environmental pollution problems. Response surface methodology (RSM) is a statistical tool for evaluating the process parameters with the fewest experiments when many factors and interactions affect desired responses for a given process. For this reason, RSM is widely used in the industrialization of active peptides obtained from proteolysis [16]. Using the effects of the enzyme substrate ratio (E/S), proteolysis time, pH, temperature, and other factors affecting yield, as well as the degree of hydrolysis (DH), activity, and other response values, proteolysis can be improved and optimized to obtain active peptides with high yield or strong activity [17,18]. Currently, ACE inhibitory peptides are prepared from stone fish protein [17], whey protein [19], lizardfish muscle protein, and similar materials using RSM [20]. However, there are few reports covering the preparation of ACE inhibitory peptides from scale gelatin using RSM. There are also few reports of the isolation and identification of these ACE inhibitory peptides. The isolation and identification of peptides is very important to factories. They can be used as markers to evaluate peptide products and then produce high-quality peptide products [14].

The purpose of this investigation was to determine the optimal conditions of neutral protease hydrolysis of scale gelatin and characterize the resulting ACE inhibitory peptides, and to prepare new ACE inhibitory peptides from the protease hydrolysis of scale gelatin.

## 2. Results and Discussion

### 2.1. Screening of Protease

**Figure 1:**
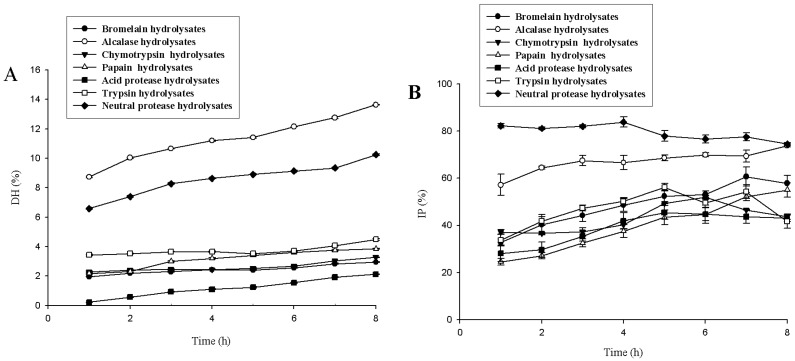
Degree of hydrolysis (DH) (**A**) and inhibition percentage (IP) (**B**) of lizardfish gelatin prepared by different proteases. Values represent means ± standard deviations (SD) of duplicate assays (*n* = 3).

The gelatin was hydrolyzed using seven proteases, and the degree of hydrolysis and ACE inhibitory activity data are shown in Figure 1. The DHs of alcalase hydrolysate and neutral protease hydrolysate gradually increased as proteolysis continued. Alcalase hydrolysate had the highest DH, and the DH increased from 8.7 ± 0.1% to 13.6 ± 0.0% (*p* < 0.05) after 1–8 h of hydrolysis. The DH of neutral protease hydrolysate was the second highest, and the DH increased from 6.6 ± 0.0% to 10.3 ± 0.1% (*p* < 0.05) after 1–8 h of hydrolysis. The DHs of other protease hydrolysates were <5%, relatively stable after 1 h of hydrolysis, and generally did not change with time. The inhibition percentage (IP) of neutral protease hydrolysates remained relatively stable during 1−4 h of hydrolysis (from 82.3 ± 0.9% to 83.8 ± 2.2%, *p* > 0.05), and then decreased to 74.6 ± 0.6% (*p* < 0.05). Therefore, neutral protease hydrolysates had the most ACE inhibitory activity and relatively higher DHs, and these were selected for further studies.

### 2.2. Effects of E/S Ratio, Hydrolysis Temperature, pH, and Time on DH and ACE Inhibitory Activity

**Figure 2:**
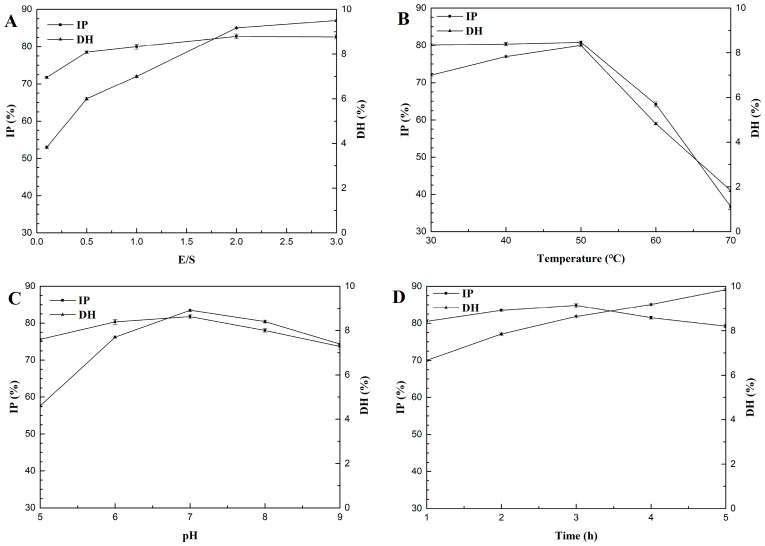
Effect of the enzyme substrate ration (E/S) (**A**), temperature (**B**), pH (**C**), and time (**D**) on the degree of hydrolysis (DH) and inhibition percentage (IP) of gelatin hydrolysates. Values represent the mean ± standard deviations (SD).

The effect of E/S from 0.1% to 3% on the DH and IP of hydrolysates was determined with 50 °C and pH 7.0 for 3 h (Figure 2A). As the enzyme concentration increased, DH and IP increased. When the enzyme concentration was >2%, DH increased slowly while IP remained the same. Hence, 2% was chosen as the center point with a 1% step change for later experiments (Table 1).

The effect of hydrolysis temperatures from 30 °C to 70 °C on the DH and IP of hydrolysates was determined with E/S 2% at pH 7.0 for 3 h (Figure 2B). When the proteolysis temperature was between 30 °C and 50 °C, DH gradually increased and IP also increased slightly. IP and DH peaked at 50 °C. When the temperature exceeded 50 °C, DH and IP declined sharply. Hence, 50 °C was chosen as the center point with 10 °C step changes for later experiments (Table 1).

The effect of pH from 5–9 on the DH and IP of hydrolysates was determined with E/S 2% at 50 °C for 3 h (Figure 2C). The highest DH and suitable IP of the hydrolysate were obtained at pH 7. Hence, pH 7 was chosen as the center point with 1 as a step change for later experiments (Table 1).

The effect of hydrolysis time from 1–5 h on the DH and IP of hydrolysates was determined with E/S 2% at 50 °C and pH 7 (Figure 2D). As hydrolysis continued, DH increased and IP first increased and then decreased. In the initial stage of proteolysis, IP increased rapidly; after 2 h of proteolysis, ACE inhibition increased slowly; after 3 h of proteolysis, ACE inhibition of the hydrolysate peaked, and then gradually decreased. This may be because the ACE inhibitor peptide is released at the beginning of proteolysis and then the activity of the hydrolysate is increased. However, when hydrolysis reaches a certain level, some ACE inhibitory peptides are further hydrolyzed by neutral protease into peptides with weaker ACE inhibitory activities. These data are consistent with previously published studies [19]. For this reason, we chose 3 h as a center point with 1 h as a step change for later experiments (Table 1).

### 2.3. Central Composite Design and Response Surface Method

The central composite design and results are shown in Table 2. We employed SAS 9.2 software (San Diego, CA, USA), using the polynomial-regression analysis method for experimental data fitting. The obtained quadratic polynomial for DH (Y_1_) and IP (Y_2_) are shown in Equations (1) and (2), respectively.

Y_1_ = 8.896667 + 0.259167 X_1_ + 0.9825 X_2_ − 1.676667 X_3_ + 0.458333 X_4_ − 1.1075 X_1_^2^ − 0.07 X_1_    X_2_ − 0.215 X_1_ X_3_ + 0.0275 X_1_ X_4_ − 0.4975 X_2_^2^ − 0.2575 X_2_ X_3_ − 0.045 X_2_ X_4_ − 2.21875 X_3_^2^ − 0.4375 X_3_ X_4_ − 0.64375 X_4_^2^ R^2^ = 0.9830                 (1)

Y_2_ = 84.54667 − 1.116667 X_1_ + 1.98 X_2_ − 3.44 X_3_ + 0.535 X_4_ − 2.024583 X_1_^2^ + 0.6775 X_1_ X_2_ −     0.175 X_1_ X_3_ − 0.3775 X_1_ X_4_ − 0.629583 X_2_^2^ − 1.49 X_2_ X_3_ − 0.4175 X_2_ X_4_ − 2.424583 X_3_^2^ − 0.175 X_3_ X_4_ + 0.412917 X_4_^2^ R^2^ = 0.9326                   (2)

As shown in Table 3, factor analysis of variance showed that the effects of pH (X_1_, *p* < 0.05), E/S (X_2_, *p* < 0.0001), temperature (X_3_, *p* < 0.0001), and time (X_4_, *p* < 0.001) on DH were all significant. However, none of the interactions between any factors other than X_3_X_4_ < 0.05 were significant (*p* > 0.05), and the quadratic terms X_1_^2^ (*p* < 0.001), X_2_^2^ (*p* < 0.01), X_3_^2^ (*p* < 0.0001), and X_4_^2^ (*p* < 0.0001) were significant. By comparing the absolute values of coefficients of the linear terms in the equation, we were able to list the factors affecting the DH in descending order of magnitude: X_3_ > X_2_ > X_4_ > X_1_. The interaction between variables on the DH were shown in Figures S1 and S3. Factor analysis of variance showed that the effects of X_1_ (*p* < 0.05), X_2_ (*p* < 0.01), and X_3_ (*p* < 0.001) on IP were significant, and that there was a significant interaction between X_2_X_3_ (*p* < 0.05). X_1_^2^ (*p* < 0.05) and X_3_^2^ (*p* < 0.001) were the only significant quadratic terms. By comparing the absolute values of coefficients of the linear terms in the equation, we were able to place the factors affecting ACE inhibition percentage in descending order of magnitude: X_3_ > X_2_ > X_1_ > X_4_. The interaction between variables on the IP were shown in Figures S2 and S4.

### 2.4. Determination of the Optimal Process Variables for the Preparation of Gelatin Peptides

Data for the partial derivative of the equation for DH appear in Equation (1). The optimal levels of the four factors were as follows: X_1_ = 0.13694, X_2_ = 1.08393, X_3_ = −0.49563, and X_4_ = 0.48944. That is, the optimal process conditions for the preparation of ACE inhibitory peptides with the DH as the indicator were as follows: pH 7.1, E/S 3.1%, temperature 45 °C, time 3 h and 30 min, and the DH was predicted to be 10% under the above conditions. By calculating the partial derivative of Equation (2), optimal levels of the four factors were obtained as follows: X_1_ = −0.04859, X_2_ = 1.19468, X_3_ = −0.33561, and X_4_ = −0.13719. That is, the optimal process conditions for the preparation of ACE inhibitory peptides with the ACE inhibition rate as the indicator were as follows: pH 7.0, enzyme dosage 3.2%, temperature 47 °C, reaction time 2 h and 50 min, and the ACE inhibition rate under the above conditions was predicated to be 86.3%. The optimal proteolysis conditions to produce ACE inhibitory activity peptides were different from the optimal proteolysis conditions to reach the highest DH. At the initial stage of proteolysis, ACE inhibitory peptides are released from the scale gelatin by protease, and the DH and ACE inhibitory activity are enhanced at this stage. As proteolysis progresses, some ACE inhibitory peptides are enzymolyzed by the protease into peptides with weaker ACE inhibitory activity. The time spent in proteolysis needed to produce the highest DH is longer than that needed to obtain the strongest ACE inhibitory activity [19]. For this reason, the optimum proteolysis parameters were pH of 7.0, enzyme dosage of 3.2%, proteolysis temperature of 47 °C, substrate concentration of 4%, and proteolysis time of 2 h and 50 min. The experimental ACE inhibitory activity under optimal conditions was 86.0 ± 0.4%, which is consistent with the predicated value of 86.3%. Under these conditions, the DH was 9.5 ± 0.4%. In addition, the gelatin peptides, prepared from lizardfish scales, reached a yield of 10.8% (dry weight). 

### 2.5. Characterization of Gelatin Peptides

As shown in Table S1, 118 peptides were identified in gelatin hydrolysates using nanoscale liquid chromatography coupled to tandem mass spectrometry (Nano-LC-MS/MS). Among these peptides, there were 26 with an Arg residue at the C-terminus, such as AGKDGMSGLPGPTGPPGPR, AGPAGASGPAGPR, and DEKSGGMPIPGPMGPMGPR. There were eight peptides with a Lys residue at the C-terminus. These were AGLPGPSGEPGK, AGPPGSDGQPGAK, GESGAPGVQGPPGPAGEEGK, GMTGSPGSPGPDGK, MTGSPGSPGPDGK, PGADGAAGGK, SGLDGAKGDSGPAGPK, and VGAPGPSGPAGPAGEK. Li et al. [21] reported that the C-terminal Arg and the guanidine on the Lys side chain and the positive charge on ε-amino groups of the active peptides play an important role in ACE inhibitory activity, and peptides containing Arg and Lys residues at the C-terminus usually have high ACE inhibitory activity. This indicates that these 34 peptides may have ACE inhibitory activity.

**Figure 3:**
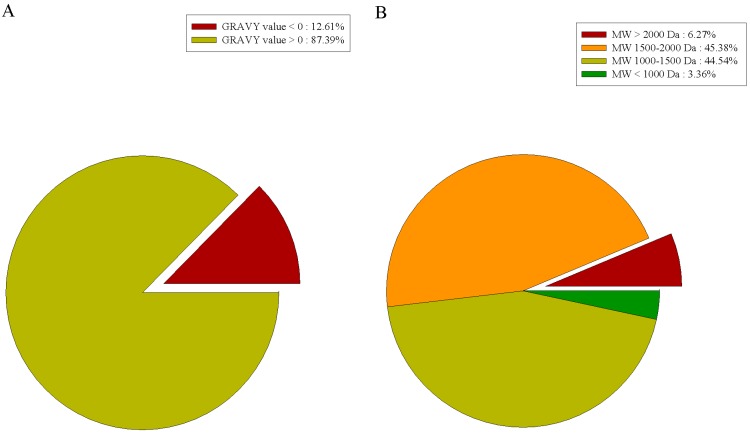
Characterization of prepared peptides. (**A**) Amino acid composition of prepared peptides; (**B**) grand average of hydropathicity (GRAVY) index value of prepared peptides.

A grand average of hydropathicity (GRAVY) index value was used to evaluate the hydrophilicity and hydrophobicity of peptides. When GRAVY > 0, the peptide is hydrophilic, and when GRAVY < 0, the peptide is hydrophobic. As shown in Figure 3A, among the 118 peptides identified in our study, there were 15 hydrophobic peptides (12.7%) and 103 hydrophilic peptides (87.3%). Hydrophilic peptides outnumbered hydrophobic peptides, and the gelatin hydrolysates might appear to be hydrophilic. This is consistent with the results reported by Liu et al., who mentioned that hydrophilic peptides are more likely to be released from the parent protein during water extraction [22].

The molecular weight (MW) distribution analysis of the identified peptides (Figure 3B) showed the MWs of all peptides to be between 815.4 and 2429.2 Da. Among them, there were 110 peptides (93.2%) with an MW below 2000 Da. Due to their low MW, these peptides cannot be degraded into amino acids and pass through the membrane barrier in the form of a whole peptide to exert their biological effects [23].

### 2.6. Stability of ACE Inhibitory Activity

**Figure 4:**
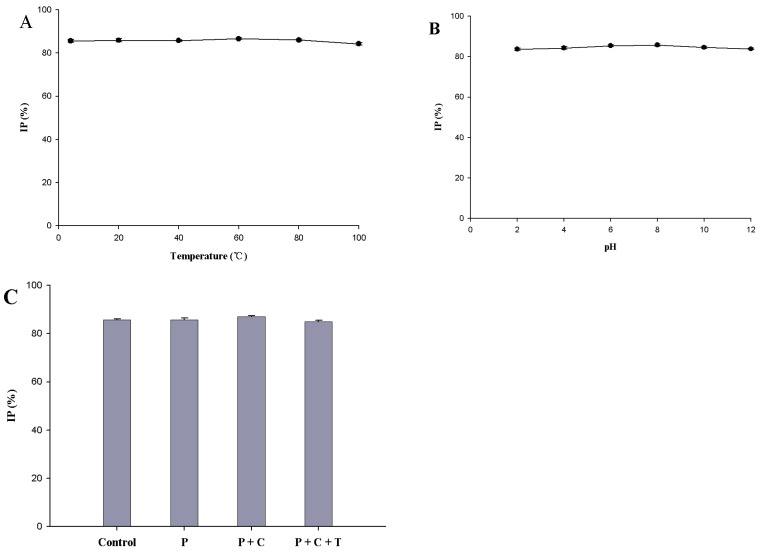
Stability of angiotensin-converting enzyme (ACE) inhibitory activity of the peptides obtained from proteolysis. (**A**) Stability of ACE inhibitory activity of peptides obtained from proteolysis after 2 h of treatment at different temperatures; (**B**) stability of ACE inhibitory activity of the peptides obtained from proteolysis after 2 h of treatment at different pH values; (**C**) stability of ACE inhibitory activity of peptides after digestion with gastrointestinal proteases. Control: peptides; P: peptides were digested with pepsin for 2 h; P + C: peptides were successively digested with pepsin for 2 h and chymotrypsin for 2 h; P + C + T: peptides were successively digested with pepsin for 2 h and chymotrypsin for 2 h and trypsin for 2 h. Values represent the mean ± standard deviations (SD).

As shown in Figure 4A,B, peptides obtained from proteolysis retained their the ACE inhibitory activity (*p* > 0.05) across different temperature and pH treatments. Data show that the ACE inhibitory activity of peptides obtained from proteolysis has thermal and pH stability. These peptides can be easily transported and stored after they are developed into a product. As shown in Figure 4C, after being treated with the digestive enzyme in the gastrointestinal tract, the peptides obtained from proteolysis maintained strong ACE inhibitory activity (*p* > 0.05), indicating that they had strong resistance to the digestive enzymes and were true inhibitor-type ACE inhibitory peptides [21]. García et al. (2013) [5] reported that ACE inhibitory peptides can exert antihypertensive effects in humans only if they are resistant to digestive enzymes and maintain antihypertensive activity in the cardiovascular and cerebrovascular systems. For these reasons, the peptides prepared in our study can enter the circulation through the gastrointestinal tract.

### 2.7. Antihypertensive Action of Gelatin Peptides in Spontaneously Hypertensive Rats (SHRs)

**Figure 5:**
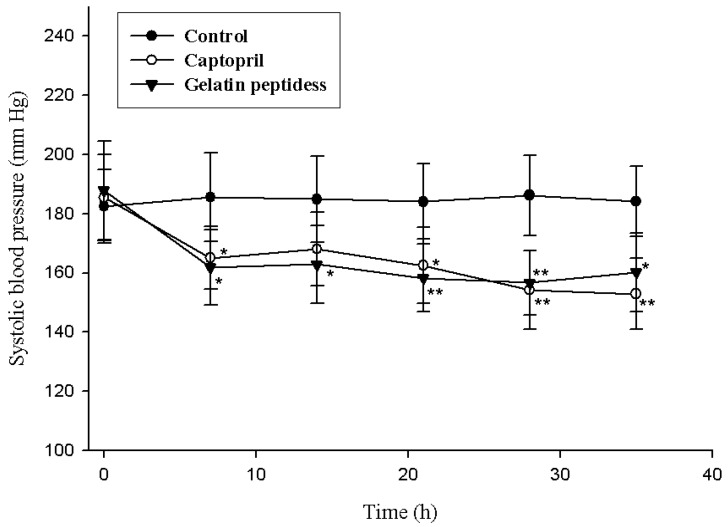
Change in systolic blood pressure (SBP) of spontaneously hypertensive rats by administering gelatin peptides. Single oral administration was performed with the dose of 2 g/kg body weight. Oral administration was performed for 35 days, and SBP was measured on days 0, 7, 14, 28, and 35. Significance of the difference from control at * *p* < 0.05, ** *p* < 0.01. Values represent means ± standard deviations (SD) of duplicate assays (*n* = 10).

As shown in Figure 5, there were no changes in systolic blood pressure (SBP) in the control group throughout the investigation period. A significant decrease in the SBP caused by 2 g/kg of gelatin peptides sample were observed from seventh day (Control_7d_: 185.6 ± 14.9 mm Hg, Sample_7d_: 161.8 ± 12.8 mm Hg, *p* < 0.05, *n* = 10). The decreases in SBP caused by gelatin peptides were more pronounced than that caused by 2 mg/kg of captopril from day 7 through day 21. This suggests that the gelatin peptides produced an antihypertensive effect in SHR at a dosage of 2 g/kg per rat in the long-term experiments.

### 2.8. Isolation and Identification of ACE Inhibitory Peptide

**Figure 6:**
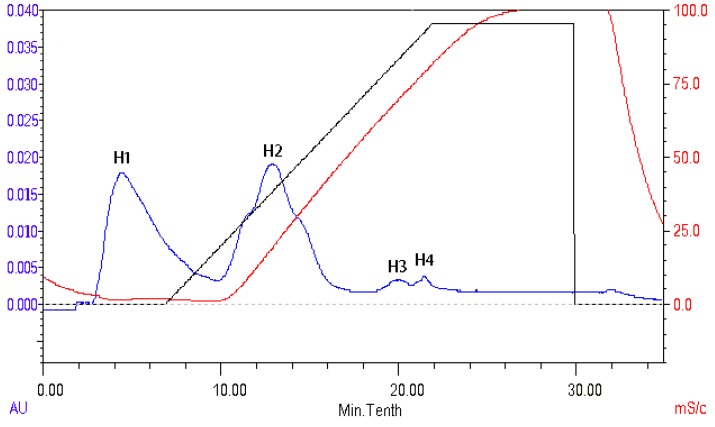
Elution profile of the gelatin hydrolysates on a HiTrapTM CaptoTM Q column.

**Figure 7:**
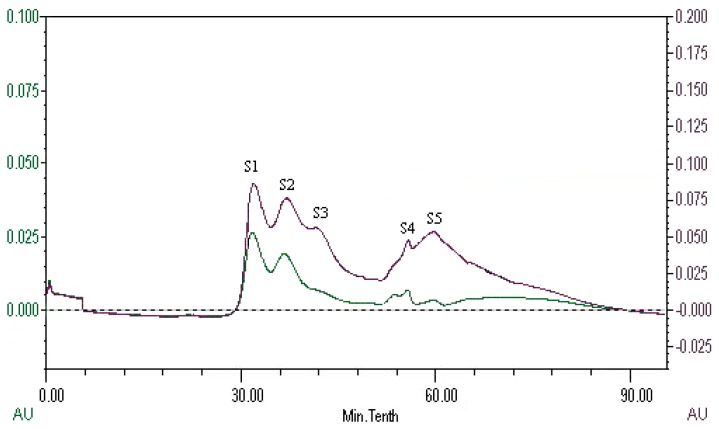
Elution profile of fraction H3 on a Sephadex G15 column.

**Figure 8:**
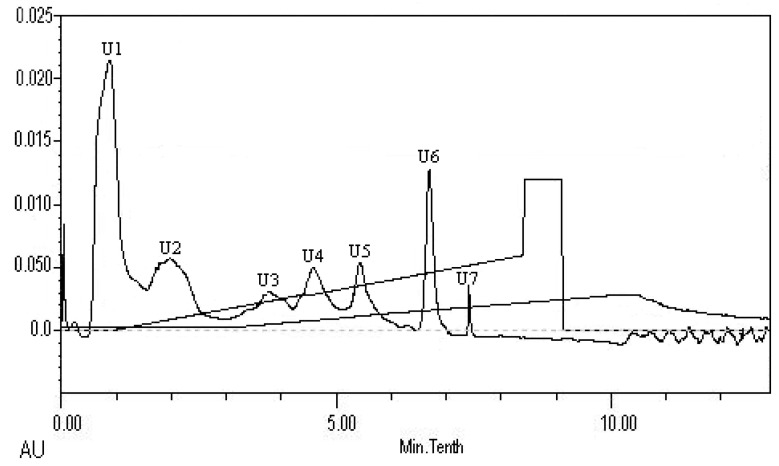
Elution profile of fraction S4 on a UNO Q1 column.

The peptides present in gelatin hydrolysates were fractionated by HiTrap^TM^ Capto^TM^ Q chromatography into four fractions (Figure 6). Fraction H3 exhibited the ACE inhibitory activity with a value of 89.4 ± 0.4%. Active fraction H3 was further purified on a Sephadex G15 chromatograph and was fractionated into five sub-fractions (Figure 7). Fraction S4 exhibited ACE inhibitory activity with a value of 87.6 ± 0.6%. As shown in Figure 8, the active fraction S4 was further separated by UNO Q1 ion-exchange chromatography to produce a purified peptide (fraction U6).

The amino acid sequence of purified peptide was found to be Ala-Gly-Pro-Pro-Gly-Ser-Asp-Gly-Gln-Pro-Gly-Ala-Lys (AGPPGSDGQPGAK). As shown in Figures S5 and S6, the purity of the peptide was 98%, and the molecular mass of peptide was 1137.3, which corresponded to its sequence. The ACE inhibitory activity of Ala-Gly-Pro-Pro-Gly-Ser-Asp-Gly-Gln-Pro-Gly-Ala-Lys (IC_50_ = 420 ± 20 μM) was higher than that of Ala-Val (IC_50_ = 956.3 μM) [24], Ala-Arg (IC_50_ = 570.8 μM) [24], and Pro-Pro-Lys (IC_50_ > 1000 μM) [1]. The difference in the ACE inhibitory activity of these peptides indicated that the C-terminal lysine had a positive effect on the polypeptide inhibitory activity [25]. The ACE inhibitory activity of Ala-Gly-Pro-Pro-Gly-Ser-Asp-Gly-Gln-Pro-Gly-Ala-Lys (IC_50_ = 420 ± 20 μM) was lower than that of Ala-Lys-Lys (IC_50_ = 3.1 μM) [21], Trp-Leu-Ala-His-Lys (IC_50_ = 77 μM) [26], and Leu-Ser-Lys (IC_50_ = 34.7 μM) [27]. These differences indicated that the low molecular weight of peptide is also an important factor of its ACE inhibitory activity [28].

## 3. Materials and Methods

### 3.1. Materials

Lizardfish (*Synodus macrops Tanaka*) was purchased from a fishery factory in Zhangzhou, China. Scales were separated manually, washed with water, and then stored in polyethylene bags at −20 °C until use. High molecular weight markers, angiotensin I-converting enzyme (from rabbit lungs), hippuryl-l-histidyl-l-leucine (Hip-His-Leu), vitamin C, pepsin, trifluoroacetic acid (TFA), and formic acid were obtained from Sigma Chemical Co. (St. Louis, MO, USA). Pepsin (3 × 10^6^ U/g) was purchased from Shanghai Generay Biological Engineering Co., Ltd. (Shanghai, China). Papain (8 × 10^6^ U/g), trypsin (3 × 10^5^ U/g), bromelain (1 × 10^6^ U/g), chymotrypsin (1 × 10^5^ U/g), neutrase (2 × 10^6^ U/g), acid protease (1 × 10^5^ U/g), and alcalase (2 × 10^6^ U/g) were purchased from Nanning Pangbo Biological Engineering Co., Ltd. (Nanning, China). LC-grade methanol and acetonitrile were purchased from Merck (Darmstadt, Germany). All other reagents used in this study were reagent grade chemicals.

### 3.2. Preparation of Scale Gelatin and Gelatin Hydrolysates

Gelatin from lizardfish scales was isolated using the method of Abdelmalek et al. [29] with slight modification. First, 1000 g of scales were added to 10 L of 0.05 mol/L NaOH, the mixture was stirred for 2 h, and then the fish scales were washed with distilled water to reach a neutral pH. The washing step was repeated three times. Pretreated scales in 8 L water were hydrolyzed using pepsin under proper proteolytic conditions: pH 1.5, E/S ratio 1:100, temperature 50 °C, and reaction time 5 h. Subsequently, pepsin was deactivated for 15 min at 100 °C and the pH was adjusted to 7.5. The mixture was centrifuged at 20,000× *g* for 30 min, and the supernatant was collected and lyophilized to produce fish scale gelatin.

The gelatin sample was added to 200 mL of pure water and enzymolyzed with bromelain (53 °C, pH 7.0), alcalase (50 °C, pH 9.0), chymotrypsin (50 °C, pH 7.5), papain (55 °C, pH 5.7), acidic protease (40 °C, pH 3.0), trypsin (37 °C, pH 8.0), and neutral protein (50 °C, pH 7.0), separately. After the enzymatic reaction was completed, the temperature was raised to 100 °C for 5 min to deactivate enzymes. The gelatin/solution ratio was 4:100 (*w*/*w*) and the E/S ratio was 1:100 (*w*/*w*). Samples were hydrolyzed for 1, 2, 3, 4, 5, 6, 7, and 8 h. Subsequently, the hydrolysate was centrifuged at 20,000× *g* for 15 min. The supernatant was lyophilized and then stored at −20 °C until use.

### 3.3. Yield of Gelatin Hydrolysates

The yield of gelatin hydrolysates was calculated based on the dry weight of the starting material. The DH was calculated using Equation (3).

Yield (%) = (Weight of lyophilized gelatin hydrolysates)/(weight of dry lizardfish scales) × 100(3)

### 3.4. DH of Gelatin Hydrolysates

The method reported by Zhang et al. [30] was modified and used to measure the DH. DH is defined as the percentage of peptide bonds cleaved [31]. Every broken peptide bond during gelatin hydrolysis could release one free amino group (−NH_2_). Taking the amount of free amino groups in gelatin hydrolysates minus the amount of free amino groups existing in gelatin, the number of peptide bonds produced by the hydrolysis of gelatin can be determined. Additionally, taking the amount of free amino groups in gelatin completely hydrolyzed by HCl minus the amount of free amino groups originally existing in gelatin, the total number of peptide bonds in gelatin can be determined. The DH of gelatin hydrolysate can be obtained by the ratio of the number of peptide bonds produced by the hydrolysis of gelatin to the total number of peptide bonds in gelatin. A mixture of 1 mL of 0.13 mg/mL gelatin hydrolysate sample solution, 1 mL of pure water, 1 mL of 0.5% ninhydrin solution, and 1 mL of 0.1% ascorbic acid solution was heated in boiling water for 15 min and allowed to cool at room temperature for 4 min. Subsequently, 7 mL of 45% (*v*/*v*) ethanol solution was added and the mixture was shaken rapidly. The optical density of the mixture was read at 580 nm and free amines in the sample were measured using a ninhydrin colorimetric curve. The DH was calculated using Equation (4).

DH (%) = (B_1_ − B_2_)/(B_3_ − B_2_) × 100 (4)

Here, B_1_ is the free amino groups in gelatin hydrolysates; B_2_ is the originally existing free amino groups in the gelatin; and B_3_ is total free amino groups in the gelatin hydrolyzed by 6 M HCl at 100 °C for 24 h.

### 3.5. Measurement of ACE Inhibition

The method reported by Cushman and Cheung (1971) [32] was modified and then used to measure ACE inhibitory activity. The sample and Hip-His-Leu were dissolved in borate buffer solutions (pH 8.3 and 100 mM), respectively. Specifically, 75 μL of 60 munits/mL ACE solution was heated at 37 °C for 15 min, and a mixture of 25 μL of 0.5 mg/mL gelatin hydrolysate/peptide sample and 225 μL of 2.5 mM Hip-His-Leu was added. This mixture was incubated at 37 °C in a water bath for 60 min, followed by the addition of 25 μL of 0.1% TFA to terminate the reaction. Hip-His-Leu is used as the substitute of angiotensin I. ACE catalyzes Hip-His-Leu to produce hippuric acid (HA). The activity of ACE was inhibited in the presence of ACE inhibitors, resulting in the reduced content of HA. The different content of the reaction-product HA was determined by reversed-phase high-performance liquid chromatography (RP-HPLC) on a CAPCELL PAK C18 MG column (4.6 × 250 mm I.D., Shiseido Co., Ltd., Tokyo, Japan). The column and eluents were maintained at 25 °C. Samples were filtered through 0.22-μm filters. Then, 10 μL of sample was loaded onto the column. The ACE inhibitory activity of the sample was determined using acetonitrile/water (25/75, *v*/*v*) containing 0.1% TFA for 10 min at a flow rate of 1 mL/min and monitored at 228 nm. ACE inhibitory activity (IP) was calculated, and A_0_ and A were chromatographic peak areas of HA with and without sample. ACE inhibitory activity was calculated using Equation (5).

IP (%) = [(A_0_ − A)/A_0_] × 100 (5)

Here, IP is the ACE inhibition percentage (%) of the sample; A_0_ and A are chromatographic peak areas of HA with and without sample. The IC_50_ value is defined as the concentration of inhibitor that can inhibit 50% of the ACE inhibitory activity.

### 3.6. Optimization Experimental Design

RSM was used to determine the optimal proteolytic conditions for the fish scale gelatin, and the experiment was optimized using a five-level four-factor Box-Behnken central composite design. pH (X_1_), E/S (%, X_2_), temperature (°C, X_3_), and time (h, X_4_) were chosen as independent variables, and the range and center point values are shown in Table 1. The DH (%, Y_1_) and ACE inhibitory activity (%, Y_2_) were selected as dependent variables for combining independent variables, as shown in Table 2. There were 27 designed experiments, and three replicates were used in each experiment to optimize the four independent variables. The substrate concentration was 4% in all experiments. Data were analyzed by multiple regressions using Statistical Analysis System software (Version 9.2, SAS Institute Inc., San Diego, CA, USA) to fit Equation (6).

Y = β_0_ + ∑β_i_ X_i_ + ∑β_ii_ X_i_^2^+ ∑β_ij_ X_i_X_j_ (6)

Here, Y is the response variables (DH or ACE inhibitory activity). β_0_ is the offset term. β_i_, β_ii_, and β_ij_ are the linear, quadratic, and interaction regression coefficient variables, respectively. X_i_ and X_j_ are independent variables.

### 3.7. Peptide Characterization by Nano-LC-MS/MS

The method reported by Liu et al. (2017) [22] was modified and used to characterized gelatin hydrolysate peptides. Samples were analyzed using a Dionex 3000 nano-LC system tandem LTQ-Orbitrap Velos Pro (Thermo Fisher Scientific, Waltham, MA, USA). Samples were re-dissolved in 0.1% TFA, and then desalted with SepPakC18. The desalted samples were dried by centrifugal concentration, and then re-dissolved in acetonitrile/formic acid/water (2/0.2/98, *v*/*v*/*v*). In brief, 5 μL of samples were loaded onto a self-made 5-μm Reprosil C18 AQ column (75 μm × 150 mm) for separation at 25 °C. The mobile phase consisted of acetonitrile/formic acid/water (2/0.2/98, *v*/*v*/*v*, buffer A) and acetonitrile/formic acid/water (80/0.2/20, *v*/*v*/*v*, buffer B); the gradient elution starting with 2% buffer B and ending with 30% buffer B lasted for 150 min. LTQ-Orbitrap operated in data-dependent acquisition mode and could automatically switch between the full scan (*m*/*z* 300–2000) in Orbitrap and the higher energy collisional dissociation (HCD) MS/MS scan in a linear ion trap. Helium was used as a collision gas for HCD. The normalized collision energy was 35% and the activation time was 30 ms. Data collection was controlled by Xcalibur 2.0.7 and Tune 2.4 software (Thermo Fisher Scientific, Waltham, MA, USA).

All MS/MS data were processed using Xcalibur (version 2.0.7.) and PEAKS 7.5 (Bioinformatics Solutions Inc., Waterloo, ON, Canada) was used. The spectra were also searched against an equal number of decoy sequences to estimate the false discovery rate, as previously described. When the variables were designed, “specified enzyme” was selected as a non-enzyme, allowing up to two restriction sites to be missed. Methionine oxidation (+15.99), protein N-terminal acetylation (+42.01), amidation (−0.98), deamidation (+0.98), formylation (+27.99), carbamylation (+43.01), methyl ester (+14.02), and carbamoyl methylation of cysteine (+57.02) were used as modifications. Default settings were used for other variables, including fragment ion tolerance set at 0.5 Da and maximum allowed mass deviation set at 6 ppm for the precursor ions after recalibration. The de novo sequence was used to identify the peptide sequence to produce the dataset, with an average local confidence (ALC) score >90%. The identified peptides were screened to create a dataset with a value of −10 logP greater than 60.

### 3.8. Stability of ACE Inhibitory Activity

#### 3.8.1. Thermal Stability

The method reported by Wang et al. [33] was used as a reference and was slightly modified. The fish scale gelatin hydrolysate was dissolved in distilled water and prepared into 1 mL of 0.4 mg·mL^−1^ solution, which was allowed to stand at 4, 20, 40, 60, 80, and 100 °C for 2 h, respectively. After samples were cooled to room temperature and pH was adjusted to 8.3 with NaOH and H_2_SO_4_, ACE inhibitory activity was measured as described in Section 3.4.

#### 3.8.2. pH Stability

The method reported by Wang et al. [33] was used as a reference and was slightly modified. The fish scale gelatin hydrolysate was dissolved in distilled water and prepared as 1 mL of a 0.4 mg·mL^−1^ solution, whose pH was adjusted to 2, 4, 6, 8, 10, and 12, respectively. These samples were allowed to stand at 37 °C for 2 h. After samples were cooled to room temperature and the pH was adjusted to 8.3 with NaOH and H_2_SO_4_, ACE inhibitory activity was measured as described in Section 3.4.

#### 3.8.3. Gastrointestinal Stability

The stability of ACE inhibitory peptide in the presence of gastrointestinal proteases was assessed in vitro using a slightly modified version of the method described by Vercruysse et al. [34]. KCl-HCl buffer solution was used to prepare 0.05 mg·mL^−1^ pepsin solution (pH 2.0), and phosphate buffer solution was used to prepare 0.05 mg·mL^−1^ trypsin solution (pH 8.0) and 0.05 mg·mL^−1^ chymotrypsin solution (pH 8.0). The gelatin hydrolysates were subjected to three digestion tests. Samples were incubated with pepsin solution with E/S 1:12 for 2 h at 37 °C. The pepsin was deactivated for 10 min at 100 °C and the pH was adjusted to 8.3. Then the mixture was centrifuged at 10,000× *g* for 10 min, and the supernatant was collected and lyophilized to measure the ACE inhibition percentage. After the pepsin treatment, samples were incubated with trypsin solution with E/S 1:12 for 2 h at 37 °C. The trypsin was deactivated for 10 min at 100 °C and the pH was adjusted to 8.3. The mixture was centrifuged at 10,000× *g* for 10 min, and the supernatant was collected and lyophilized to measure the ACE inhibition percentage. After the treatment of pepsin and trypsin, samples were incubated with chymotrypsin solution with E/S 1:12 for 2 h at 37 °C. The chymotrypsin was deactivated for 10 min at 100 °C and the pH was adjusted to 8.3, the mixture was centrifuged at 10,000× *g* for 10 min, and the supernatant was collected and lyophilized. The ACE inhibitory activity was measured as described in Section 3.4.

### 3.9. Antihypertensive Action of Gelatin Peptides in SHRs

SHRs (10 weeks old, male, SPF, 200–290 g body weight) with tail SBP over 180 mmHg were obtained from Beijing Vital River Laboratory Animal Technology Co., Ltd. (Beijing, China). SHRs were housed individually in steel cages in a room kept at 24 °C under controlled lighting from 8:00 to 20:00, and fed a standard laboratory diet. SBP was measured by the tail-cuff method with an Non invasive blood pressure (NIBP) system (Powerlab ML125, AD Instrument, Shanghai, China). Scale gelatin peptides prepared from lizardfish were dissolved in saline and administrated orally at a dose of 2 g/kg body weight. Captopril was used as a positive control and was administrated the same way as the scale gelatin peptides at a dose of 2 mg/kg body weight. Control rats were administrated orally with the same volume of saline solution. Oral administration was performed for 35 days. The SBP was measured on days 0, 7, 14, 28 and 35.

### 3.10. Isolation and Identification of ACE Inhibitory Peptide

One milliliter of gelatin hydrolysates was loaded onto an HiTrap^TM^ Capto^TM^ Q ion-exchange column (7 × 25 mm), equilibrated with 20 mM Tris-HC buffer (pH 8.0), and eluted with a linear gradient of NaCl (0–1 M) in the same buffer at a flow rate of 1 mL/min. The absorbance of the eluent was monitored at 228 nm. Fractions showing ACE inhibitory activity were collected and lyophilized. One ACE inhibitory activity fraction was re-dissolved in super-pure water. After that, the solution was further purified on a self-made Sephadex G15 gel column (2.5 × 15 cm), and eluted with super-pure water at a flow rate of 1 mL/min. The absorbance of the eluent was monitored at 228 nm and 280 nm. The injection of the sample was 1 mL. The fraction showing ACE inhibitory activity was lyophilized. The ACE inhibitory activity fraction was re-dissolved in super-pure water. After that, 1 mL of solution was loaded onto a UNO Q1 ion-exchange column (7 × 35 nm), equilibrated with 50 mM Tris-HC buffer (pH 8.0), and eluted with a linear gradient of NaCl (0–2 M) in the same buffer at a flow rate of 4 mL/min. The absorbance of the eluent was monitored at 228 nm. The purified peptide was collected and lyophilized for further use.

The amino acid sequence of the purified peptide was determined by automated Edman degradation using a Shimadzu PPSQ-33A protein sequencer. The purity of peptide was determined by RP-HPLC on a SinoChrom ODS-BP C18 column (4.6 × 250 mm) using the linear gradient of acetonitrile (6–94%) containing 0.1% TFA in the same buffer at a flow rate of 1 mL/min, while monitoring the absorbance at 220 nm. The molecular mass of peptide was determined by Waters ZQ2000 mass.

## 4. Conclusions

RSM was used to optimize proteolytic conditions for gelatin hydrolysates. The optimal conditions were as follows: pH 7.0, E/S 3.2%, temperature 47 °C, and proteolysis time 2 h and 50 min. The experimental ACE inhibition rate of the gelatin peptides obtained under the optimal conditions was 86.0 ± 0.4%, which is consistent with the predicated value of 86.3%. The gelatin peptides, prepared from lizardfish scales, reached a yield of 10.8% (dry weight). There were 118 peptides identified from the gelatin peptides. Among them, 87.3% were hydrophilic peptides and 93.2% had an MW of less than 2000 Da. There were 26 peptides with C-terminal Arg residues and eight peptides with C-terminal Lys residues, and these 34 peptides may have ACE inhibitory activity. Gelatin peptides show strong high stability under exposure to extremes of high temperature and pH, and the digestive enzymes in the gastrointestinal tract, suggesting that gelatin peptides may pass through the membrane barrier in the form of whole peptides to be directly absorbed by the body. In the long-term experiments, the gelatin peptides showed an antihypertensive effect in SHR at a dosage of 2 g/kg per rat. The decrease in SBP caused by gelatin peptides was greater than that caused by 2 mg/kg of captopril from days 7–21. Although the dosage of gelatin peptides was larger than that of the captopril, gelatin peptides were prepared from food resources and could be eaten daily without toxic side effects. In addition, a new ACE inhibitory peptide was isolated from gelatin hydrolysates and identified as AGPPGSDGQPGAK, with an IC_50_ value of 420 ± 20 μM. This indicates that our prepared ACE inhibitory peptides may be suitable for further development into health products and drugs raw materials. 

## Figures and Tables

**Table 1:** Variable values in the central composite design for gelatin hydrolysates.

Independent Variables	Variable	Coded Levels		
		−1	0	1
X_1_	pH	6	7	8
X_2_ (%)	E/S	1	2	3
X_3_ (°C)	Temperature	40	50	60
X_4_ (h)	Time	2	3	4

**Table 2:** Experimental designs and results of the gelatin hydrolysates.

Standard Order	Code Level of Variable				Response Value	
	X_1_	X_2_ (%)	X^3^ (°C)	X_4_ (h)	DH (%)	IP (%)
1	−1	−1	0	0	6.1 ± 0.1 ^a^	81.9 ± 0.8 ^a^
2	−1	1	0	0	8.0 ± 0.1 ^b^	84.7 ± 0.3 ^b^
3	1	−1	0	0	6.6 ± 0.1 ^c^	76.5 ± 0.4 ^c^
4	1	1	0	0	8.2 ± 0.1 ^d^	82.0 ± 1.0 ^a^
5	0	0	−1	−1	6.6 ± 0.1 ^c^	84.5 ± 0.9 ^b^
6	0	0	−1	1	8.6 ± 0.1 ^e^	85.3 ± 0.8 ^b,d^
7	0	0	1	−1	4.2 ± 0.2 ^f^	78.9 ± 0.9 ^e^
8	0	0	1	1	4.5 ± 0.0 ^g^	79.0 ± 0.0 ^e^
9	−1	0	0	−1	6.4 ± 0.1 ^h^	82.7 ± 0.4 ^a,f^
10	−1	0	0	1	7.3 ± 0.1 ^i^	85.5 ± 0.8 ^b,g^
11	1	0	0	−1	6.6 ± 0.1 ^c^	81.1 ± 1.2 ^a^
12	1	0	0	1	7.6 ± 0.0 ^j^	82.4 ± 1.0 ^a^
13	0	−1	−1	0	6.4 ± 0.1 ^h^	83.8 ± 1.2 ^b,f,h^
14	0	−1	1	0	3.4 ± 0.0 ^k^	74.3 ± 1.0 ^i^
15	0	1	−1	0	9.1 ± 0.1 ^l^	85.7 ± 0.9 ^b,g^
16	0	1	1	0	5.1 ± 0.1 ^m^	82.1 ± 1.3 ^a^
17	−1	0	−1	0	6.8 ± 0.1 ^n^	84.9 ± 0.5 ^b^
18	−1	0	1	0	3.9 ± 0.1 ^o^	77.1 ± 0.4 ^c^
19	1	0	−1	0	8.2 ± 0.1 ^d^	84.8 ± 0.1 ^b^
20	1	0	1	0	4.4 ± 0.1 ^g^	76.3 ± 0.7 ^c^
21	0	−1	0	−1	6.7 ± 0.0 ^c,n^	82.8 ± 0.7 ^a,h^
22	0	−1	0	1	7.4 ± 0.1 ^i^	84.3 ± 0.5 ^b^
23	0	1	0	−1	8.7 ± 0.1 ^e,p^	86.5 ± 0.4 ^d,g^
24	0	1	0	1	9.2 ± 0.1 ^l^	86.3 ± 0.2 ^d,g^
25	0	0	0	0	8.8 ± 0.1 ^p,q^	84.1 ± 0.4 ^b^
26	0	0	0	0	8.9 ± 0.1 ^q^	84.3 ± 0.4 ^b^
27	0	0	0	0	9.0 ± 0.1 ^l^	85.2 ± 0.3 ^b^

X_1_: pH; X_2_: E/S; X_3_: Temperature; X_4_: Time. Values represent the mean ± standard deviations (SD). ^a–q^ Values with different letters in the same column of DH are significant different at the *p* < 0.05 level. ^a–g^ Values with different letters in the same column of IP are significant different at the *p* < 0.05 level.

**Table 3:** Analysis of variance (ANOVA) for degree of hydrolysis (DH) (Y_1_) and inhibition percentage (IP) (Y_2_).

Source	Mean Square		*F*-Value		*p*-Value	
	DH	IP	DH	IP	DH	IP
Model	5.555053	19.6431	49.53511	11.86095	<0.0001 ***	<0.0001 ***
X_1_	0.096671	0.371496	7.187278	9.0352	0.0200 *	0.0109 *
X_2_	0.096671	0.371496	103.2931	28.40672	<0.0001 ***	0.0002 ***
X_3_	0.096671	0.371496	300.8151	85.74476	<0.0001 ***	<0.0001 ***
X_4_	0.096671	0.371496	22.47859	2.07395	0.0005 ***	0.1754
X_1_X_2_	0.167439	0.643451	0.174776	1.108634	0.6833	0.3131
X_1_X_3_	0.167439	0.643451	1.648777	0.073968	0.2234	0.7903
X_1_X_4_	0.167439	0.643451	0.026974	0.344194	0.8723	0.5683
X_2_X_3_	0.167439	0.643451	2.365045	5.362187	0.1500	0.0391 *
X_2_X_4_	0.167439	0.643451	0.072229	0.421	0.7927	0.5287
X_3_X_4_	0.167439	0.643451	6.827175	0.073968	0.0227 *	0.7903
X_1_^2^	0.145007	0.557245	58.33257	13.20017	0.0001 ***	0.0034 **
X_2_^2^	0.145007	0.557245	11.7709	1.276482	0.0050 **	0.2806
X_3_^2^	0.145007	0.557245	234.121	18.93139	<0.0001 ***	0.0009 ***
X_4_^2^	0.145007	0.557245	19.70871	0.549077	0.0008 ***	0.4729
Residual	0.112144	1.656115				
Lack-of-fit	0.131366	1.914652	8.193295	5.268234	0.1136	0.1700
Pure error	0.016033	0.363433				
Cor total	5.667197	21.299215				

* *p* *<* 0.05; ** *p* *<* 0.01; *** *p* *<* 0.001.

## Supplementary Materials

The following are available online at http://www.mdpi.com/1660-3397/16/7/228/s1, Figure S1: Response surfaces plots showing the interaction between variables on the DH; Figure S2: Response surfaces plots showing the interaction between variables on the IP; Figure S3: Contour plots showing the interaction between variables on the DH; Figure S4: Contour plots showing the interaction between variables on the IP; Figure S5: Molecular mass of AGPPGSDGQPGAK performed by Waters ZQ2000 mass spectrometry analysis; Figure S6: Chromatogram on a SinoChrom ODS-BP C18 column of AGPPGSDGQPGAK; Table S1: Peptides identified from lizardfish scale gelatin hydrolysates.
