# Supplementary material for: Processing Optimization and Characterization of Angiotensin-Ι-Converting Enzyme Inhibitory Peptides from Lizardfish (Synodus macrops) Scale Gelatin
Source: Mar Drugs. 2018 Jul 4;16(7):228. doi: 10.3390/md16070228 (PMC6071053; doi:10.3390/md16070228)
Supplement: Supplementary file 1 [file marinedrugs-16-00228-s001.pdf]

Supplementary Materials

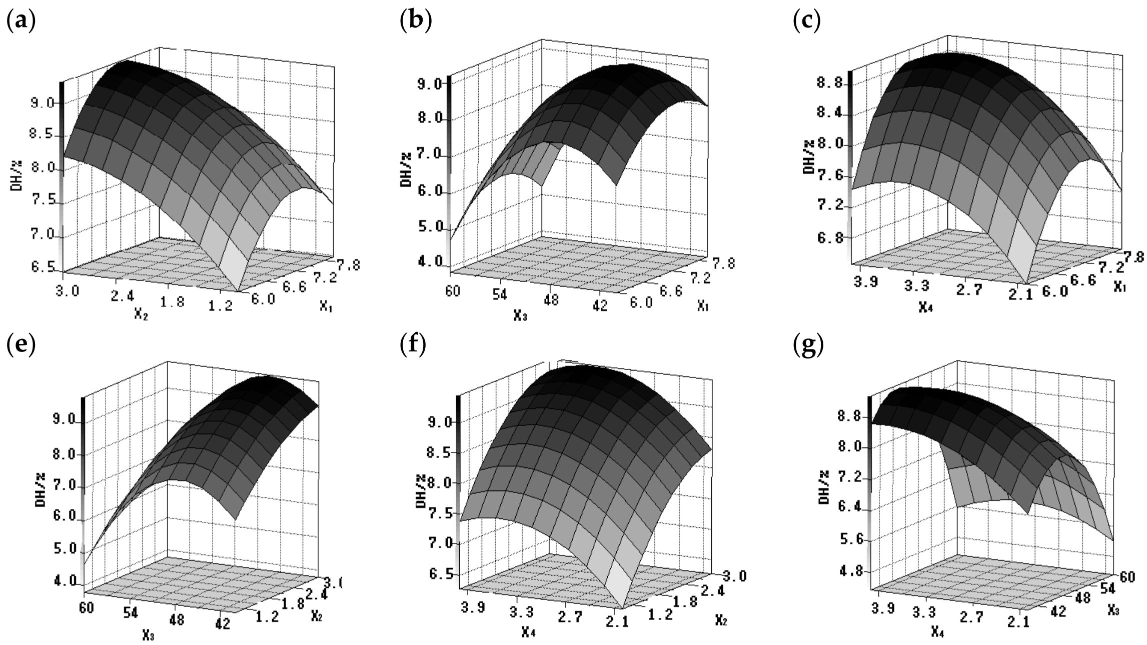

Figure S1. Response surfaces plots for the effects of variables on the diameter of the degree of hydrolysis : (a): X_1 and X_2; (b): X_1 and X_3; c: X_1 and X_4; d: X_2 and X_3; e: X_2 and X_4; f: X_3 and X_4.

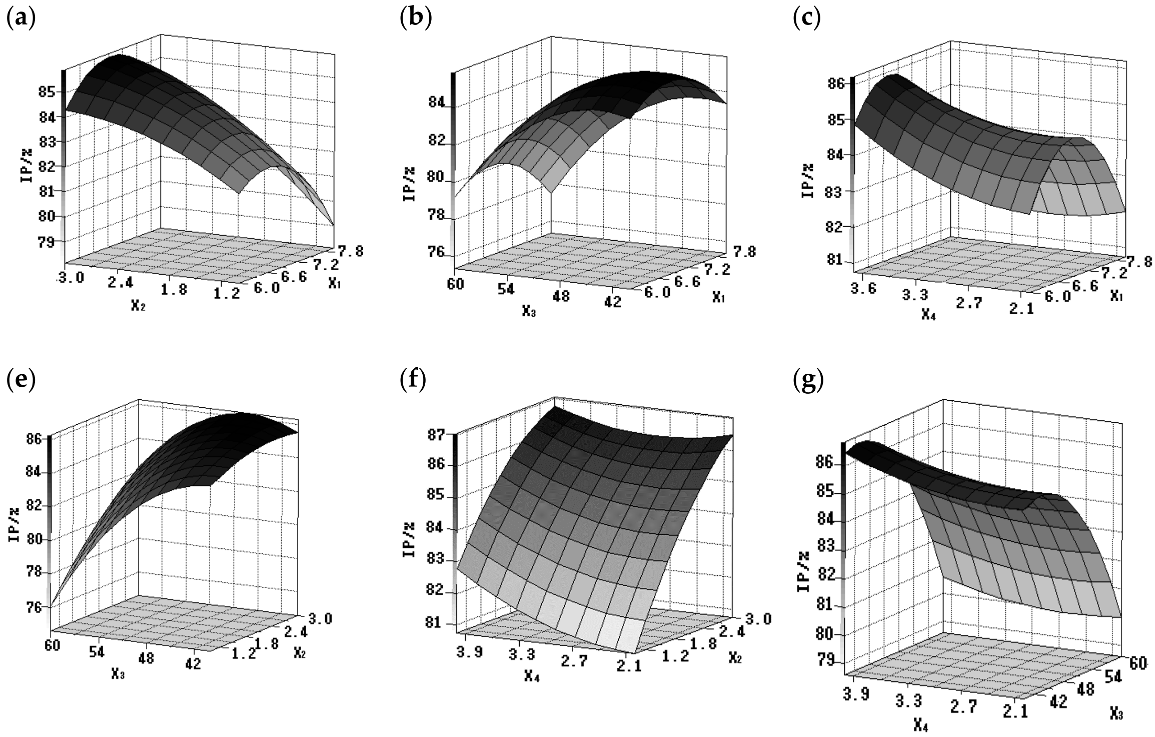

Figure S2. Response surfaces plots for the effects of variables on the diameter of the inhibition percentage (IP) : (a): X_1 and X_2; (b): X_1 and X_3; c: X_1 and X_4; d: X_2 and X_3; e: X_2 and X_4; f: X_3 and X_4.

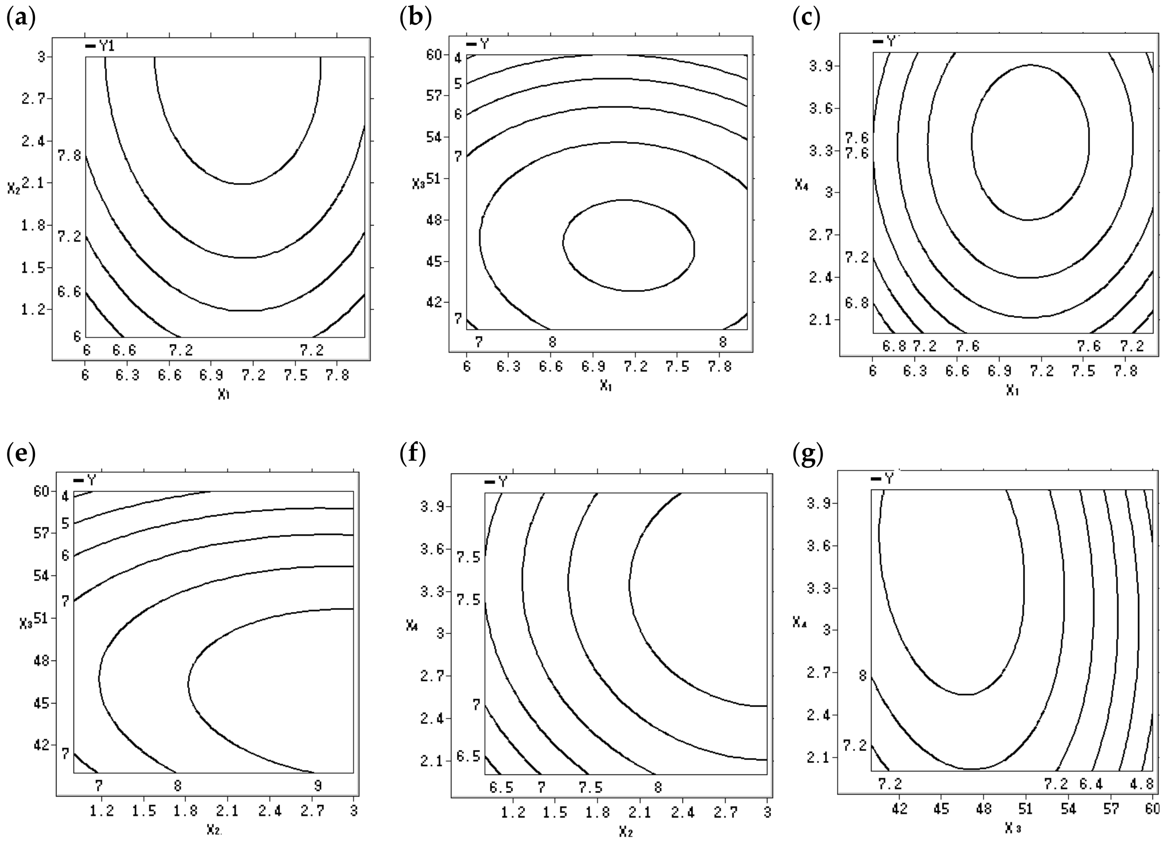

Figure S3. Contour plots for the effects of variables on the diameter of the degree of hydrolysis (DH) : (a): X_1 and X_2; (b): X_1 and X_3; c: X_1 and X_4; d: X_2 and X_3; e: X_2 and X_4; f: X_3 and X_4.

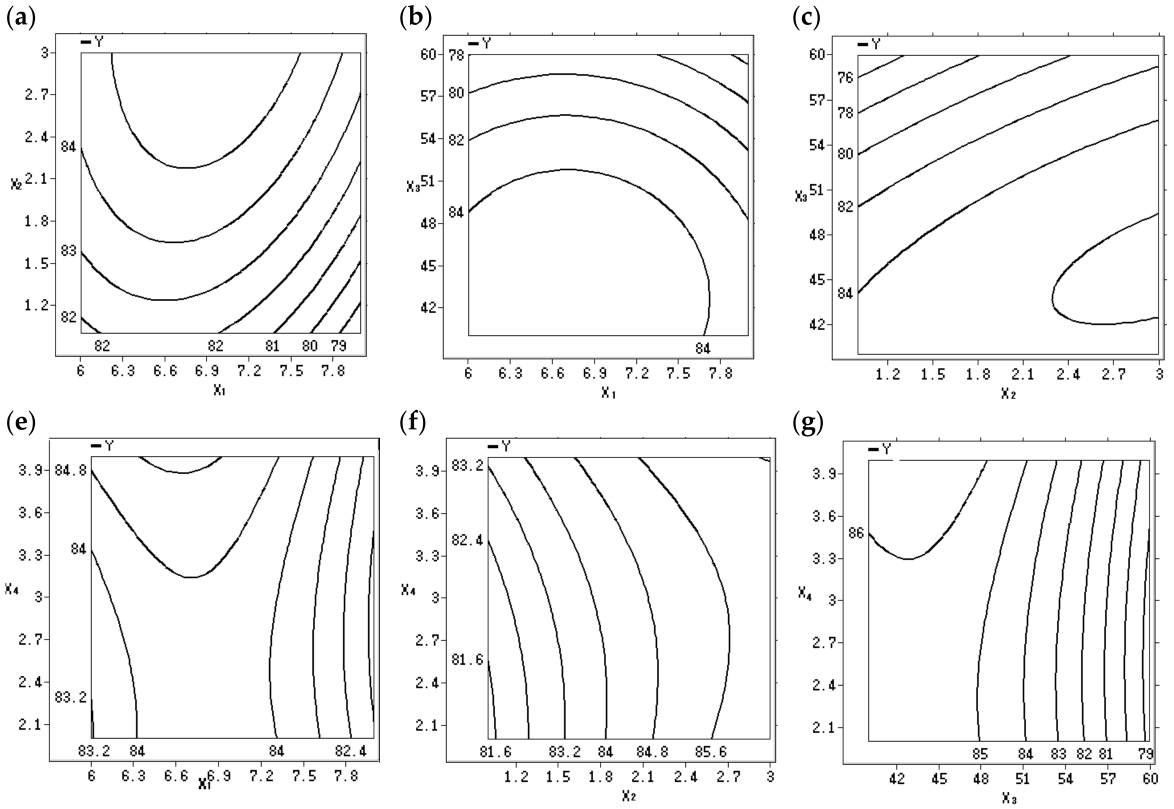

Figure S4. Response surfaces plots for the effects of variables on the diameter of the inhibition percentage (IP) : (a): X_1 and X_2; (b): X_1 and X_3; c: X_1 and X_4; d: X_2 and X_3; e: X_2 and X_4; f: X_3 and X_4.

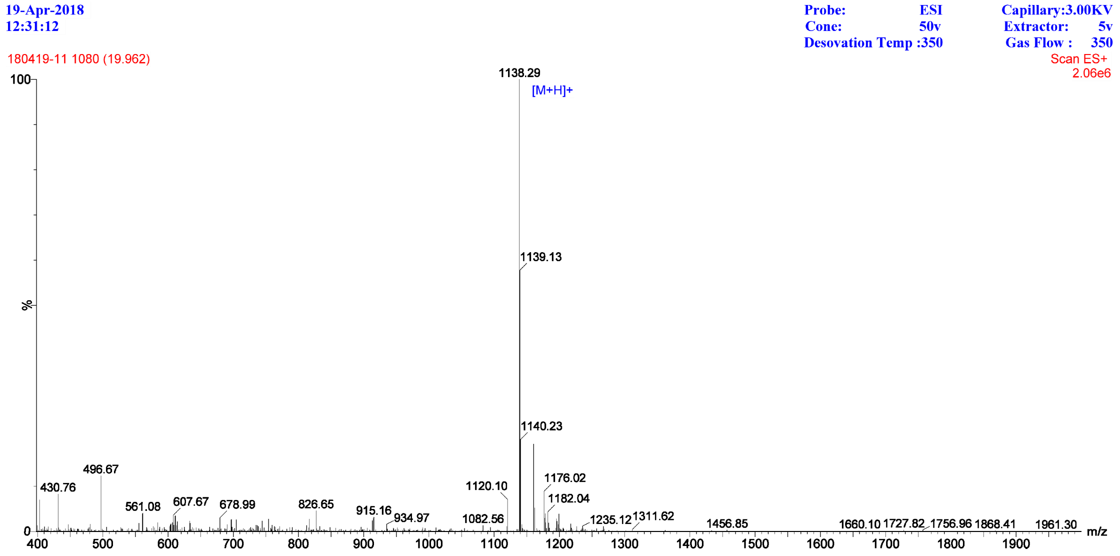

Figure S5. AGPPGSDGQPGAK, *m/z* 1137.29 performed by Waters ZQ2000 mass spectrometry analysis.

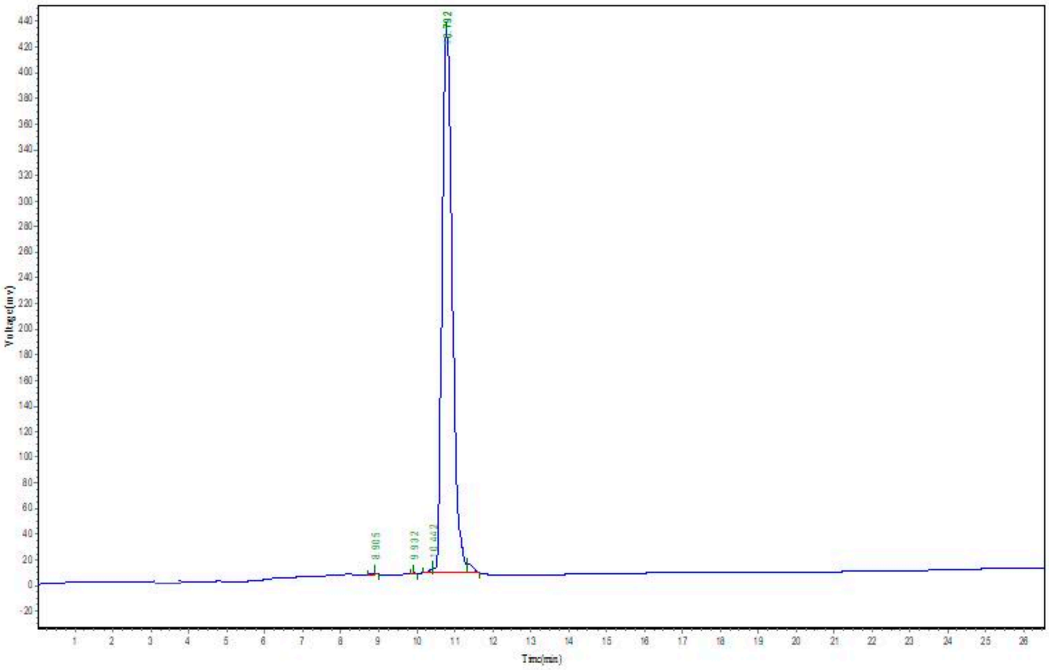

Figure S6. Chromatogram on a SinoChrom ODS-BP C18 column of the AGPPGSDGQPGAK .

Table S1. Peptides identified from lizardfish scales gelatin

No.	Peptide	GRAVY	Mass
1	AGKDGMSGLPGPTGPPGPR	-0.858	1763.8624
2	AGLPGPSGEPGK	-0.75	1123.5509
3	AGLPGPSGEPGKQGAPGS	-0.772	1620.7743
4	AGPAGASGPAGPR	-0.346	1064.5363
5	AGPPGSDGQPGAK	-1.115	1137.5414
6	AVGETGKPGEQGLPGEA	-0.718	1611.7739
7	DDKSPAMPVPGPM	-0.669	1340.6105
8	DDKSPAMPVPGPMGPM	-0.55	1625.7251
9	DEKSGGMPIPGPM	-0.723	1357.6006
10	DEKSGGMPIPGPMGPM	-0.594	1642.7153
11	DEKSGGMPIPGPMGPMG	-0.582	1699.7368
12	DEKSGGMPIPGPMGPMGPR	-0.842	1968.8855
13	DEKSPAMPVPGPM	-0.669	1354.6261
14	DEKSPAMPVPGPMGPM	-0.55	1639.7408
15	DEKSPAMPVPGPMGPMG	-0.541	1696.7622
16	DEKSPAMPVPGPMGPMGPR	-0.805	1965.911
17	DEKSPAMPVPGPSGPM	-0.719	1595.7323
18	DGMNGIPGPIGPPGPR	-0.662	1530.7612
19	DGMNGIPGPIGPPGPRG	-0.647	1603.7776
20	DGMNGLPGPIGPPGPR	-0.706	1530.7612
21	DGMNGLPGPIGPPGPRG	-0.688	1603.7776
22	DGMSGLPGPTGPPGPR	-0.863	1491.714
23	DGMSGLPGPTGPPGPRG	-0.835	1564.7303
24	DGVSGLPGPTGPPGPR	-0.719	1489.7524
25	DHSKSSGPPVPGPM	-1.007	1503.6064
26	DHSKSSGPPVPGPMGPM	-0.835	1788.7212
27	DKSPAMPVPGPM	-0.433	1225.5835
28	DKSPAMPVPGPMGPM	-0.353	1510.6982
29	EKSGGMPIPGPMGPM	-0.4	1527.6884
30	EKSPAMPVPGPM	-0.433	1239.5991
31	EKSPAMPVPGPMGPM	-0.353	1524.7139
32	FAGPPGADGQAGAR	-0.45	1300.616
33	FTGPPGEPGEAGASGPMGPR	-0.755	1883.8472
34	GASGPAGPRGPAGSA	-0.38	1208.5897
35	GDRGESGPAGASGAPGPPGAPGPVGPA	-0.544	2239.0618
36	GEPGLIGPPGPPGPAGDI	-0.244	1612.8096
37	GEQGPAGPSGAPGPR	-1.14	1333.6375
38	GEQGPAGSAGPAGPR	-0.913	1307.6218
39	GERGPPGLPGPPGL	-0.743	1299.6935
40	GESGAPGVQGPPGPAGEEGK	-1.005	1792.8226
41	GETGAAGENGTPGAMGPR	-0.817	1658.7318

42	GFAGLPGPSGEPGKQGAPGS	-0.575	1824.8641
43	GFDEKSGGMPIPGPMGPM	-0.394	1846.8052
44	GGLGPQGGPGEDGERG	-1.319	1438.6437
45	GGPGEPGPSGPSGPRG	-1.275	1377.6272
46	GGPGPQGPSGPSGPR	-1.333	1319.6218
47	GGPGPQGPSGPSGPRG	-1.275	1376.6432
48	GGPRGPPGDAGRA	-1.208	1179.5745
49	GIQGPVGLPGPAGPI	0.46	1344.7401
50	GKDGMSGLPGPTGPPGP	-0.8	1536.7242
51	GKDGMSGLPGPTGPPGPR	-1.006	1676.8303
52	GKDGMSGLPGPTGPPGPRG	-0.974	1749.8468
53	GKDGMSGMPGPIGPLGPR	-0.522	1722.8545
54	GLTGPIGLPGPAGAT	0.5	1293.6929
55	GMPGPAGPPGPTGANGDKGESGS	-0.974	2025.8698
56	GMPGPAGPPGPTGANGDKGESGSFGPA	-0.733	2398.0496
57	GMSGLPGPTGPPGPR	-0.687	1392.6819
58	GMSGLPGPTGPPGPRG	-0.669	1449.7034
59	GMTGSPGSPGPDGK	-1.043	1259.5452
60	GNVGPAGPAGPLG	0.1	1062.5458
61	GPAGSAGSAGKDGMSGLPGPTGPPGPR	-0.633	2390.1284
62	GPAGSAGSPGKDGMSGLPGPTGPPGPR	-0.759	2374.1335
63	GPAGSSGSPGKDGMNGLPGPIGPPGPR	-0.763	2429.1758
64	GPGGERGPNGPMG	-1.292	1197.5197
65	GPRGPPGPPGSSGPQG	-1.35	1400.6796
66	GPSGNIGLPGMTGPQGEAGRE	-0.743	1997.9113
67	GPSGPAGPPGARGD	-0.971	1207.5581
68	GPTGPVGAPGKDGDVGAQ	-0.572	1594.7587
69	GRTGPIGMPGARG	-0.515	1241.6299
70	GSAGPGGERGPPGPMGPPGL	-0.705	1743.8362
71	GSPGKDGMNGEPGPIGPPGPDG	-1.141	1988.8898
72	GSPGKDGMNGIPGPIGPPGPR	-0.843	1972.9789
73	GSPGKDGMNGLPGPIGPPGPR	-0.876	1972.9789
74	IAVPGPMGPMG	0.755	1025.5038
75	IGFPGPKGPAGE	-0.392	1141.5767
76	IGLPGMTGPQ	0.16	985.4902
77	IGLPGMTGPQGEA	-0.038	1242.5914
78	IGLPGMTGPQGEAGR	-0.36	1456.698
79	IGLPGMTGPQGEAGRE	-0.556	1585.7406
80	IGMPGMTGPQ	-0.03	1003.4467
81	IGMPGMTGPQGEAGRE	-0.675	1602.713
82	KGDNGISGPTGPLGPIGPPGL	-0.314	1921.9873
83	KSGGMPIPGPMGPM	-0.179	1412.6614
84	LGAPGKDGDVGAPGAPGPAGPSGE	-0.467	2061.9602
85	LGEPGRVGPA	-0.26	967.5087

86	LTGPIGLPGPAGAT	0.564	1236.6714
87	LTGPIGLPGPAGATGD	0.25	1408.7197
88	MGFPGPKGPGGD	-0.792	1131.5018
89	MGPTGPVGAPGKD	-0.508	1198.5652
90	MGPTGPVGAPGKDGDVGAQ	-0.442	1725.7992
91	MKAPMSAFSAV	0.827	1138.5515
92	MPVPGPMGPM	0.27	1012.4544
93	MTGSPGSPGPDGK	-1.092	1202.5237
94	PAMPVPGPMGPMG	0.192	1251.5814
95	PAMPVPGPMGPMGPR	-0.24	1490.7196
96	PGADGAAGGK	-0.52	815.3773
97	PGLAGPVGEPGKQGPSGPS	-0.689	1683.8579
98	PGLPGPSGEPGKQGAPGS	-0.961	1604.7794
99	PIGLPGPAGAT	0.473	965.5181
100	PIGLPGPAGATGD	0.1	1137.5665
101	PKGELGPIGPLGPPGPPGE	-0.653	1838.9413
102	QGGAGPSGERGPPGPMGPPGL	-0.838	1871.8948
103	QGPGGPGGERGPPGPM	-1.275	1462.6622
104	QGPSGPGGERGPPGPMGPPG	-1.24	1800.8213
105	QGPSGPGGERGPPGPMGPPGL	-1	1913.9053
106	RGADGNVGPAGPAGPLG	-0.312	1461.7324
107	RGSPGPDGNNGPAGPVG	-1.094	1504.7018
108	SGGMPIPGPMGPM	0.108	1284.5665
109	SGLDGAKGDSGPAGPK	-0.887	1412.6895
110	SPAMPVPGPMGPM	0.162	1267.5763
111	SPAMPVPGPMGPMGPR	-0.275	1593.7466
112	TGPPGEPGEAGASGPMGPR	-0.942	1736.7787
113	VGAPGPSGPAGPA	0.062	1049.5142
114	VGAPGPSGPAGPAGEK	-0.438	1363.6731
115	VGERGPPGPMGPPGL	-0.54	1416.7183
116	VGERGPPGPMGPPGLSGAPGEAG	-0.504	2074.9741
117	VMGPTGPVGAPGKDGDVGAQ	-0.21	1824.8676
118	YDDKSPAMPVPGPMGPM	-0.594	1788.7885

*The grand average of hydropathicity (GRAVY) index value was used to evaluate the hydrophilic and hydrophobic character of the identified peptides
